# Comparative effects of ball sports on executive function subdomains in children and adolescents: a network meta-analysis

**DOI:** 10.3389/fpsyg.2026.1830417

**Published:** 2026-06-10

**Authors:** Tongyan Zhang, Muyan Zhang, Yang Wang, Anastasiia V. Kabachkova, Zixuan Luo, Rong Ma

**Affiliations:** 1 Faculty of Physical Education, Tomsk State University, Tomsk, Russia; 2 Institute of Integrative Healthcare, Siberian State Medical University, Tomsk, Russia; 3 School of Physical Education, Shanxi Normal University, Taiyuan, Shanxi, China

**Keywords:** adolescents, ball sports, children, cognitive flexibility, executive function, inhibitory control, working memory

## Abstract

**Objectives:**

Ball sports interventions have been shown to yield positive effects on executive functions (EF). The aim of this study is to use network meta-analysis (NMA) to evaluate the differences in the impact of different ball sports on the subdomains of EF among children and adolescents.

**Methods:**

Five databases, including PubMed, Cochrane, Embase, Web of Science, and Scopus, were searched up to November 2025 to identify randomized controlled trials measuring the effect of different ball sports on the subdomains of EF among children and adolescents. Paired analyses and network meta-analyses were conducted using the random-effects model.

**Results:**

This study included 12 studies with five ball sports interventions. Ball sports showed domain-specific effects on EF in children and adolescents. The surface under the cumulative ranking curve (SUCRA) reveals that football may be a potentially effective intervention for improving the accuracy rate of inhibitory control (SUCRA = 76.69%). For working memory, ball sports did not consistently enhance accuracy, with the control condition showing the highest ranking (SUCRA = 80.93%). In contrast, tennis exhibited the greatest likelihood of improving reaction time (SUCRA = 99.99%). Table tennis may be a potentially effective intervention for improving reaction time of cognitive flexibility (SUCRA = 99.97%). Sensitivity analyses restricted to typically developing samples revealed notable changes in network structures and SUCRA rankings for inhibitory control accuracy, inhibitory control reaction time, and cognitive flexibility accuracy.

**Conclusion:**

Different ball sports demonstrated varying effects across executive function subdomains. However, the findings for inhibitory control and cognitive flexibility were highly dependent on sample composition and lacked robustness, whereas those for working memory were relatively stable. Due to limited evidence and high heterogeneity, the results should be interpreted with caution.

**Systematic review registration:**

https://www.crd.york.ac.uk/PROSPERO/view/CRD420251038836.

## 1 Introduction

During the growth and development of children and adolescents, executive function (EF) plays a vital role (Collins and Koechlin, 2012). EF is a set of higher-order cognitive processes that involve the top-down control processes triggered when planning, organizing, and monitoring complex goal-directed behaviors (Donnelly et al., 2016). It includes three main subdomains: inhibitory control (IC) (Munakata et al., 2011), working memory (WM) (Baddeley, 2012), and cognitive flexibility (CF) (Baddeley, 2012). These three subdomains cooperate with each other and have their own functional emphases, jointly influencing the daily life, academic performance, and social interaction development of children and adolescents. Attentional processes may serve as a fundamental mechanism underlying the development of EF (Oberauer, 2019). Selective attention, sustained attention, and attentional shifting are closely associated with the development of IC, WM, and CF during childhood and adolescence (Commodari et al., 2025; Foti et al., 2026). Developmental psychology research indicates that the maturation of attentional control abilities facilitates the progressive improvement of higher-order EF in children and adolescents (Petersen and Posner, 2012). In real-world contexts, children constantly allocate attentional resources, suppress irrelevant stimuli, maintain sustained attention, and flexibly adjust attentional focus in response to environmental changes (Foti et al., 2026). Consequently, activities that place high demands on attentional regulation and adaptive information processing may be particularly beneficial for the development of EF in children and adolescents.

Physical exercise intervention is an important strategy for promoting the development of EF in children and adolescents (World Health Organization, 2020). Both acute and chronic exercise have been associated with improvements in specific EF subcomponents. The underlying mechanisms may involve increased cerebral blood flow, enhanced neuroplasticity, and modulation of prefrontal cortical activity (Fontes et al., 2020; Hötting and Röder, 2013; Tomoto et al., 2023). Furthermore, sport contexts typically require continuous attentional regulation, rapid information processing, and adaptive behavioral control, all of which may further facilitate the development of EF-related abilities. However, the type of exercise and the differences in cognitive and attentional demands may influence the magnitude and specificity of these benefits. Open-skill activities characterized by dynamic and unpredictable environments may be more beneficial for IC in children and adolescents, whereas closed-skill aerobic exercises involving repetitive and stable movement patterns may confer greater advantages for WM (Zhu et al., 2023). In addition, mind–body exercises, active play, and structured physical activity programs have also been shown to improve specific cognitive functions (Tao et al., 2025). Among numerous forms of exercise, ball sports may involve greater attentional and cognitive demands due to their movement complexity, environmental dynamism, and social interaction characteristics. Team ball sports such as basketball (Li et al., 2024) and football (Matos et al., 2023) require participants to continuously allocate and update attentional resources across teammates, opponents, spatial information, and tactical contexts, while simultaneously inhibiting task-irrelevant responses. In contrast, racket sports such as table tennis (Tsai, 2009) and tennis (Ishihara et al., 2017) rely more heavily on rapid visuospatial attention, anticipatory processing, and fast attentional shifting in response to high-speed ball trajectories and continuously changing competitive situations. These ball sport-specific attentional demands may exert differential effects across EF subdomains, thereby leading to distinct cognitive adaptations. Moreover, ball sports may positively influence EF through enhanced prefrontal neural activity, improved cerebral hemodynamics, and exercise-induced neuroplasticity.

Existing meta-analyses have typically examined physical exercise or open-skill activities as a whole, with limited differentiation between specific types of ball sports and a lack of systematic comparisons across EF subdomains (Tao et al., 2025; Zhu et al., 2023). Studies based on cross-sectional data have identified an association between ball sports and EF (Wang et al., 2025); however, due to the inherent limitations of the study design, causal inferences cannot be established. Although multiple randomized controlled trials (RCTs) have reported beneficial effects of ball sports on EF (Ishihara et al., 2017; Tsai, 2009), these studies have generally focused on a single sport or assessed only one EF subcomponent. Consequently, comprehensive comparative evidence regarding multiple ball sports and multiple EF subdomains remains lacking. The relative advantages and effectiveness of different ball sport interventions across EF subdomains, therefore, remain unclear and warrant further investigation.

Network meta-analysis (NMA) is a novel analytical method that combines direct evidence with indirect evidence and is capable of simultaneously comparing the effects of more than two interventions in a single analysis (De Laat, 2017). Meanwhile, it can rank different interventions for a single outcome and display the probability of the relative efficacy of each intervention, which helps guiden treatment decisions. Therefore, this study used NMA to compare the relative effects of different ball sports on the three core EF subdomains, utilizing both direct and indirect evidence to inform exercise-based cognitive intervention strategies for youth.

## 2 Methods

### 2.1 Registration

The present systematic review and NMA was carried out in accordance with the Preferred Reporting Items for Systematic Reviews and Meta-Analyses (PRISMA) guidelines (Page et al., 2021). Moreover, its protocol has already been registered on the International prospective register of systematic reviews (CRD420251038836).

### 2.2 Search strategy

Systematic searches were conducted in Embase, PubMed, Cochrane Library, Web of Science, and Scopus for studies investigating the effect of ball sports on EF in children and adolescents up to November, 2025. The references of included studies were also tracked. According to the PICOS (Participant, Intervention, Comparison, Outcome, Study) principles, the strategy of combining “keywords” and “free terms” was adopted for searching, including adolescents, children, ball sports, Inhibitory control, working memory, cognitive flexibility, etc. See Supplementary Table S1 for a detailed example of the Web of Science search strategy.

### 2.3 Selection of studies and eligibility criteria

After the searched studies were imported into EndNote 20, and duplicate records were removed, two reviewers (T and Y) independently screened the literature using the following inclusion and exclusion criteria. Inclusion criteria: (1) Study type: RCTs and human studies; (2) Study population: children and adolescents aged between 6 and 18 years old, with no history of concussion and free from injuries that could impair motor function (e.g., fractures, severe sprains); (3) Intervention types: All ball sports were included, and each study was required to cover at least one ball sport item. The control group needed to be effectively controlled, for example, by means of being put on a waiting list, receiving no intervention, or watching videos; (4) Outcome measures: It is required that the research reports should encompass at least one outcome indicator related to the subdomains of EF. Specifically, this this requirement entails the inclusion of the accuracy rates and reaction times corresponding to the task paradigms of IC, WM, and CF, respectively. Exclusion criteria: (1) Duplicates; (2) Systematic reviews, meta-analyses, and conference papers; (3) Animal studies; (4) Inaccessible full text and relevant information. EndNote software was used for literature management. Duplicate records were automatically identified and removed using the deduplication function. The search and filtering functions were then applied to exclude animal studies, conference proceedings, systematic reviews, and meta-analyses based solely on titles or abstracts, thereby improving the accuracy and efficiency of the study selection process. Literature retrieval and screening were conducted independently by two reviewers (T and Y). In case of any discrepancies, a consensus was reached through discussion or consultation with a third reviewer.

### 2.4 Data extraction

The data of eligible studies was independently extracted and organized into Excel 2019 by two reviewers (T and M), including (1) Basic information: Title, first author, year of publication, and study type; (2) Participant information: Country, average age, sample size, body mass index (BMI), and gender ratio; (3) Information on exercise interventions: Type of exercise, number, weeks, frequency, and duration. (4) Outcome measures: Accuracy and reaction time performance in IC tasks, WM tasks, and CF tasks. Data extraction was performed independently by two reviewers (T and M), and any discrepancies were resolved through discussion or consultation with a third reviewer to reach a consensus.

### 2.5 Risk of bias and strength of evidence assessment

The risk of bias in the included RCTs was assessed employing the Cochrane risk-of-bias tool for randomized trials (RoB 2). This evaluates the quality and bias of the included outcomes across five domains: (1) bias arising from the randomization process, (2) bias due to deviations from intended interventions, (3) bias due to missing outcome data, (4) bias in measurement of the outcome, and (5) bias in selection of the reported result (Sterne et al., 2019). The risk-of-bias judgments for each domain were “low risk of bias,” “some concerns,” or “high risk of bias.” The certainty of the comparative results was assessed with the Confidence in Network Meta-Analysis (CINeMA) framework. Evaluations were conducted across the following six domains: (1) within-study bias, (2) indirectness, (3) reporting bias, (4) imprecision, (5) inconsistency, and (6) heterogeneity. Each domain was rated as “no concerns,” “some concerns,” or “major concerns” based on the severity of bias. The overall confidence rating was categorized into four levels: “very low,” “low,” “moderate,” and “high.” The quality assessment was independently performed by two reviewers (T and M). In case of any discrepancies, a consensus was reached through discussion or consultation with a third reviewer.

### 2.6 Subgroup and sensitivity analyses

When significant statistical or clinical heterogeneity was identified, subgroup and sensitivity analyses were further conducted to explore potential sources of heterogeneity and inconsistency. Subgroup analyses were stratified according to country, age group, population type, exercise modality, and session duration in order to evaluate the potential influence of these factors on intervention effects. Sensitivity analyses were performed using a leave-one-out approach to assess the robustness of the pooled effect estimates. In addition, to examine the potential effect-modifying influence of clinical samples on the overall effect estimates and their impact on the generalizability of the findings, a restricted sensitivity analysis was conducted, including only typically developing children and adolescents.

### 2.7 Data synthesis and analysis

Traditional pairwise meta-analysis was conducted using a random effects model in Stata 18. The results are expressed as standardized mean difference (SMD) with a 95% confidence interval (CI). The *I^2^* statistic was employed to assess the heterogeneity of treatment effects. When the *I^2^* ≥ 50%, the heterogeneity was regarded as significant (Melsen et al., 2014). A Random-effects network within a Bayesian framework model was established using the Markov chain Monte Carlo method in R Studio (Hamra et al., 2013). Model parameters were set as follows: four chains were used (Bois, 2013; Hamra et al., 2013), with a step size of 1, an annealing count of 20,000, and a simulation iteration of 50,000 (Kaltenthaler et al., 2011). Based on the different ball sports intervention measures included in the studies, a network graph was constructed to visually display the relationships among these intervention measures. The Deviance Information Criterion (DIC) was used to assess model fit. A dDIC ≤ 10 was considered to indicate no significant global inconsistency (Veroniki et al., 2013). For this study, as it was observed that the network diagram did not possess a closed loop, node-splitting analysis was not carried out. The surface under the cumulative ranking curve (SUCRA) was adopted to rank the interventions and handle the uncertainty in their effects. The value of SUCRA ranges from 0 to 100%, and a larger value indicates that the intervention measure is more effective (Salanti et al., 2011). For publication bias, visual inspection using funnel plots and evaluation through Egger’s test (Irwig et al., 1998) were conducted. Publication bias was considered absent when the symmetry of the funnel plot was inspected visually or when the *p* ≥ 0.05.

## 3 Results

### 3.1 Study selection

**Figure 1:**
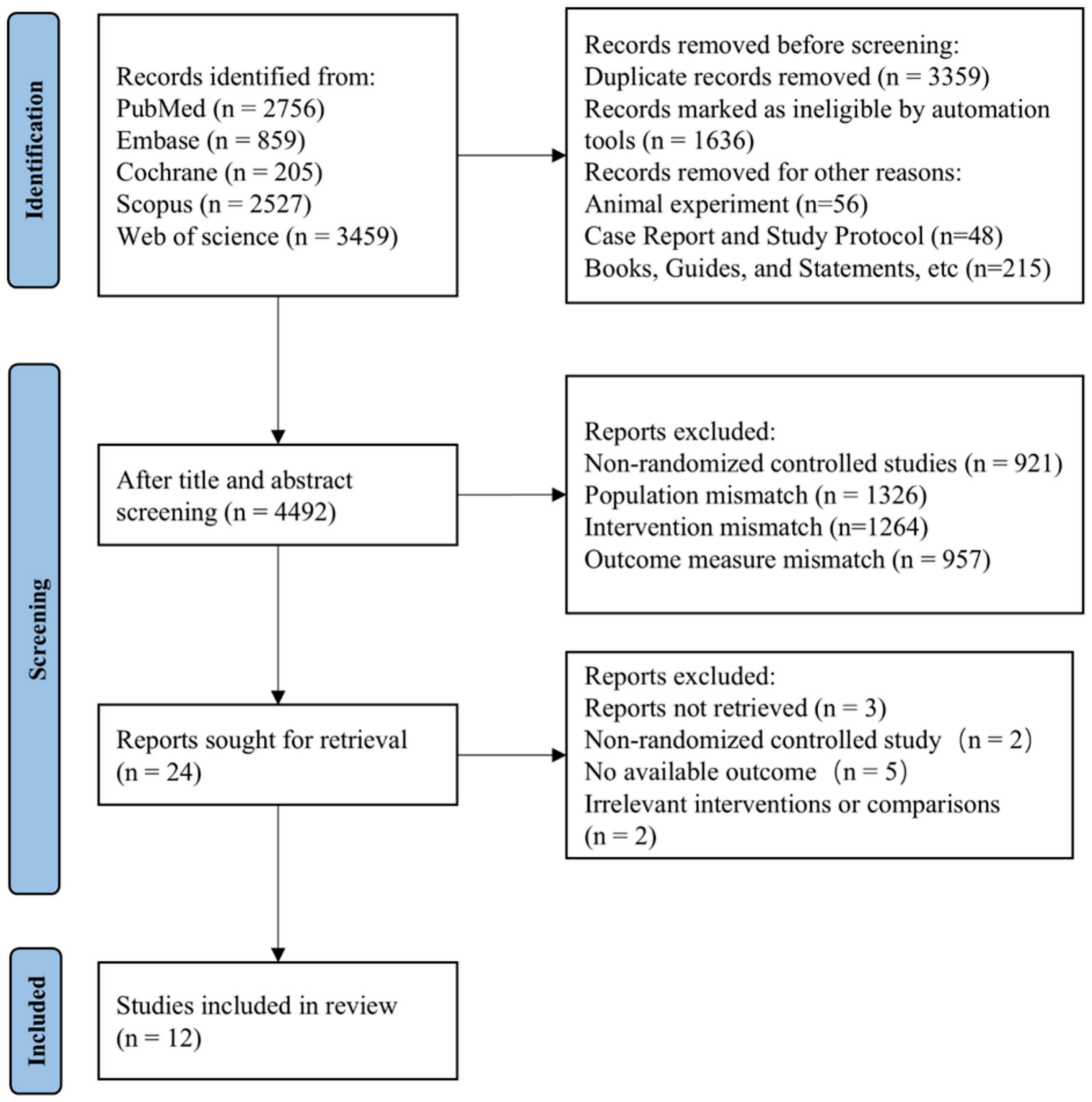
PRISMA flow diagram of the study selection process.

A total of 9,806 articles were retrieved from database searches and imported into EndNote 20. After removing 3,359 duplicate records, 6,423 articles were excluded based on title and abstract screening due to reasons such as reviews, conference papers, animal studies, and obviously irrelevant publications, leaving 24 articles for further full-text screening. After reading the full text, an additiona12 articles were excluded for reasons such as reports not retrieved, non-RCT studies, no available outcome, irrelevant interventions or comparisons, and irrelevant participants. Finally, 12 articles were included in the review and NMA analysis. The PRISMA flowchart is presented in Figure 1.

### 3.2 Characteristics of the included studies

A total of 12 studies were included, involving 751 children and adolescents, with 423 in the experimental group and 328 in the control group. The mean age was 11.81 ± 2.65 years. These studies were conducted in various countries, including the United States of America, Denmark, France, and Japan (1 study each); the United Kingdom (2 studies); and China (6 studies). The experimental group used five ball sports: badminton (1 study), basketball (2 studies), football (5 studies), table tennis (3 studies), and tennis (1 study), whereas the control group continued with their regular daily activities. The basic characteristics of the included studies are presented in Table 1.

**Table 1:** Characteristics of studies included in the review.

Author/year	Country	Intervention measure			Participants				Outcome		
		Exercise type	Number	Weeks | frequency | duration	Status	Age (mean ± SD)	BMI (mean ± SD)	Gender (M/F)	IC	WM	CF
Chang et al. (2022)	China	Table tennis	16	12 weeks | 3 times | 60 min	ADHD	8.31 ± 1.30	NA	NA	NA	NA	Wisconsin Card Sorting Test (ACC)
		Control	16			8.38 ± 1.31					
Tse et al. (2019)	China	Basketball	19	12 weeks | 2 times | 45 min	ASD	10.11 ± 1.20	19.86 ± 2.71	14/5	Go/Nogo task (ACC)	NA	NA
		Control	21			9.81 ± 1.17	18.76 ± 2.27	18/3			
Cooper et al. (2018)	UK	Basketball	39	1 time | 60 min	Healthy	12.30 ± 0.70	18.80 ± 2.60	NA	Stroop task (ACC, RT)	Sternberg paradigm (ACC, RT)	NA
		Control	39								
Dong et al. (2024)	USA	Football	38	70 times | 30 min	Healthy	18.34 ± 0.58	20.23 ± 2.89	27/11	Flanker task (ACC, RT)	2-back task (ACC, RT)	odd task (ACC, RT)
		Control	32			18.34 ± 0.48	20.72 ± 2.60	20/12			
Ishihara et al. (2017)	Japan	Tennis	71	1 time | 50 min	Healthy	9.75 ± 0.25	16.27 ± 0.33	32/39	Stroop task (ACC, RT)	2-back task (ACC, RT)	Local–global Task (ACC, RT)
		Control	10			9.10 ± 0.50	16.90 ± 0.60	6/4			
Ji et al. (2022)	China	Football	33	6/9 weeks | 3 times | 60 min	ASD	13.1 ± 2.97	22.4 ± 2.55	17/16	Flanker task (ACC) Stroop color-word test (ACC)	NA	NA
		Control	33			12.8 ± 2.69	21.8 ± 2.76	18/15			
Pan et al. (2017)	China	Table tennis	11	12 weeks | 2 times | 70 min	ASD	9.68 ± 1.61	18.56 ± 3.73	11/0	NA	NA	Wisconsin Card Sorting Test (ACC)
		Control	11			8.49 ± 1.76	17.02 ± 3.66	11/0			
Pinelli et al. (2026)	France	Badminton	60	10 times | 90 min	Healthy	11.06 ± 0.42	NA	NA	Stroop task (ACC, RT) Go/Nogo task (ACC, RT)	NA	NA
		Control	52								
Lind et al. (2019)	Denmark	Football	27	1 time | 20 min	Healthy	11.9 ± 0.38	19.31 ± 1.07	16/11	Flanker task (ACC, RT)	NA	NA
		Football	27			11.8 ± 0.38	18.90 ± 0.92	16/11			
		Control	27			11.7 ± 0.38	18.78 ± 0.79	16/11			
Tsai (2009)	China	Table tennis	14	10 weeks | 3 times | 50 min	DCD	9.53 ± 0.53	NA	NA	Endogenous Posner task (ACC, RT)	NA	NA
		Control	14			9.47 ± 0.29					
Williams et al. (2020)	UK	Football	36	1 time| 90 min	Healthy	12.60 ± 0.50	NA	20/16	Stroop task (ACC, RT)	Sternberg Paradigm (ACC, RT)	NA
		Control	36								
Wen et al. (2021)	China	Football	32	1 time | 40 min	ASD	10.90 ± 0.30	17.90 ± 0.20	15/17	Go/Nogo task (ACC, RT)	2-back task (ACC, RT)	NA
		Control	37			10.90 ± 0.40	18.10 ± 0.80	20/17			

ACC, accuracy; ADHD, attention deficit hyperactivity disorder; ASD, autism spectrum disorder; CF, cognitive flexibility; DCD, developmental coordination disorder; F, female; IC, inhibitory control; M, male; NA, not available; RT, reaction time; SD, standard deviation; UK, United Kingdom; USA, United States of America; WM, working memory.

### 3.3 RoB 2 quality evaluation

Among the 12 included studies, 50% had “low risk of bias”, 41.7% had “some concerns,” and 8.3% had “high risk of bias.” Regarding different domains of bias, the bias arising from the randomization process was categorized as “low risk” in 50% of studies, “some concerns” in 41.7% of studies, and “high risk” in 8.3% of studies. Deviations from intended interventions resulted in a bias categorized as “low risk” in 75% of studies and “some concerns” in 25% of studies. Bias due to missing outcome data was classified as “low risk” in 100% of studies. Bias in measurement of the outcome was identified as “low risk” in 83.3% of studies and “some concerns” in 16.7% of studies. Bias in selection of the reported results was rated as “low risk” in 91.7% of studies and “some concerns” in 8.3% of studies. The primary sources of risk were as follows: 3 studies mentioned random allocation using random number tables or computer programs, and 2 studies described random grouping by independent personnel, while the remaining 8 studies did not provide detailed descriptions of the randomization method. Blinding participants in exercise-related studies had some difficulties; therefore, only 2 studies used single blinding, and the remaining studies lacked detailed descriptions. All studies provided evidence that the results were not influenced by missing data, 6 studies reported that participants were not included in the analysis due to reasons like incomplete data, poor training performance, illness, injury, or transferring to another school. All studies adopted appropriate outcome measurement methods, avoiding the selective reporting of results. Supplementary Figure S1 represents the ROB diagram. According to the CINeMA assessment, most pairwise comparisons were found to have low confidence levels. The CINeMA results are in Supplementary Table S2.

### 3.4 Pairwise meta-analysis and NMA

**Figure 2:**
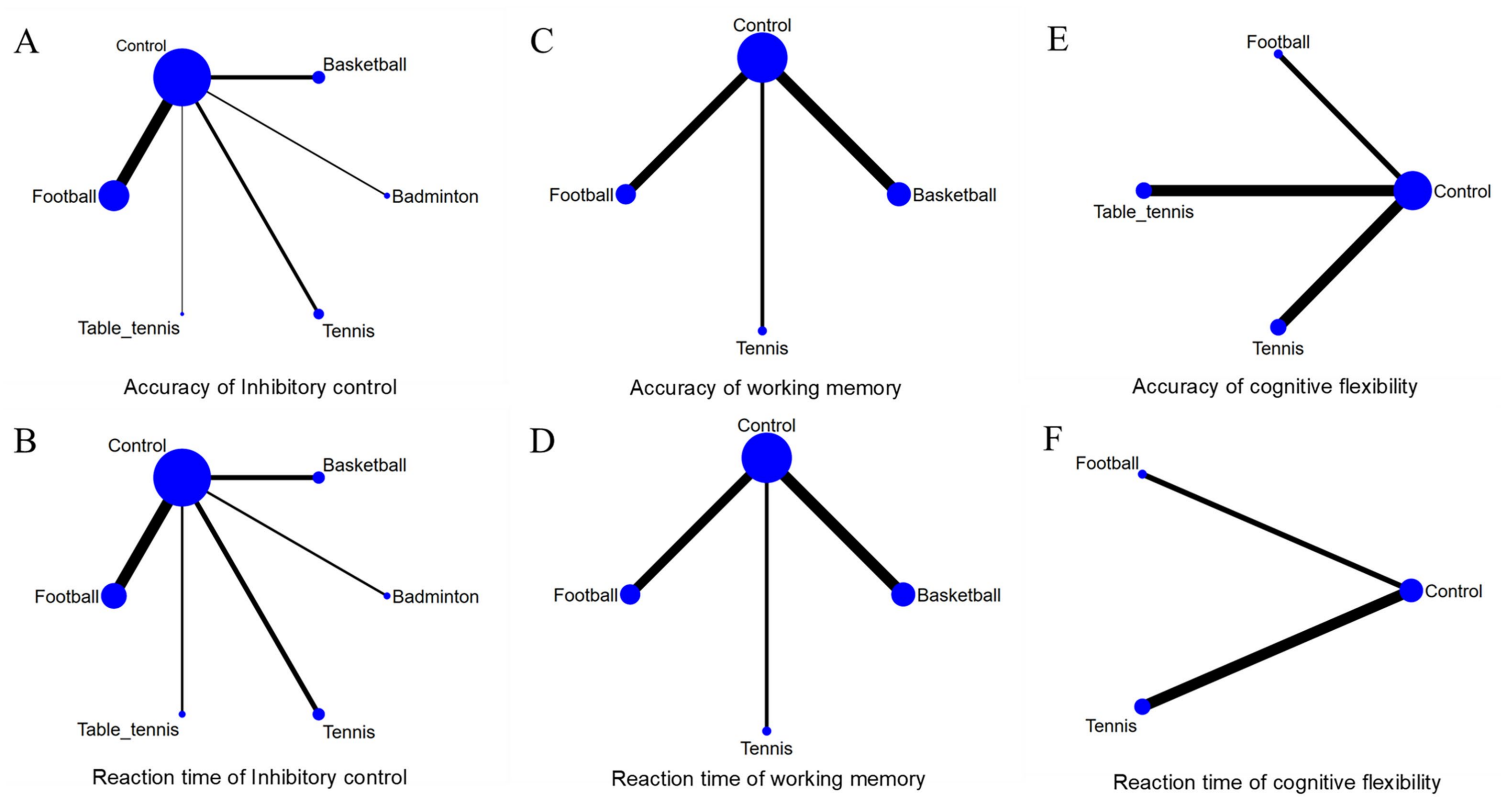
Network graph. Each node represents one Training type. The lines between the dots indicate a direct comparison between the two modes of motion, with thicker lines for more studies and thinner lines for fewer studies. **(A)** Accuracy of Inhibitory control, **(B)** reaction time of inhibitory control, **(C)** accuracy of working memory, **(D)** reaction time of working memory, **(E)** accuracy of cognitive flexibility, **(F)** reaction time of cognitive flexibility.

The included 12 studies discussed five different ball sports interventions: badminton, basketball, football, table tennis, and tennis. Figures 2A–F shows the network structure diagram that illustrates the relationships between these interventions. In this figure, the thickness of the lines within the diagram can mirror the quantity of pairwise comparisons among the interventions. Moreover, the size of the circles, which represent the interventions is proportional to the number of participants included in each of the interventions. The difference in DIC values between the consistency model and the inconsistency model was less than 5, and the *I^2^* values of all outcomes were less than 25%, indicating the absence of global inconsistency. Global inconsistency and heterogeneity of each outcome in the Supplementary Table S3. Owing to the limited and sparse evidence base, no closed-loop network structures were identified for any outcome in the present study. Consequently, comparisons based primarily on indirect evidence were associated with substantial uncertainty and should be interpreted with considerable caution.

#### 3.4.1 Inhibitory control performance

**Figure 3:**
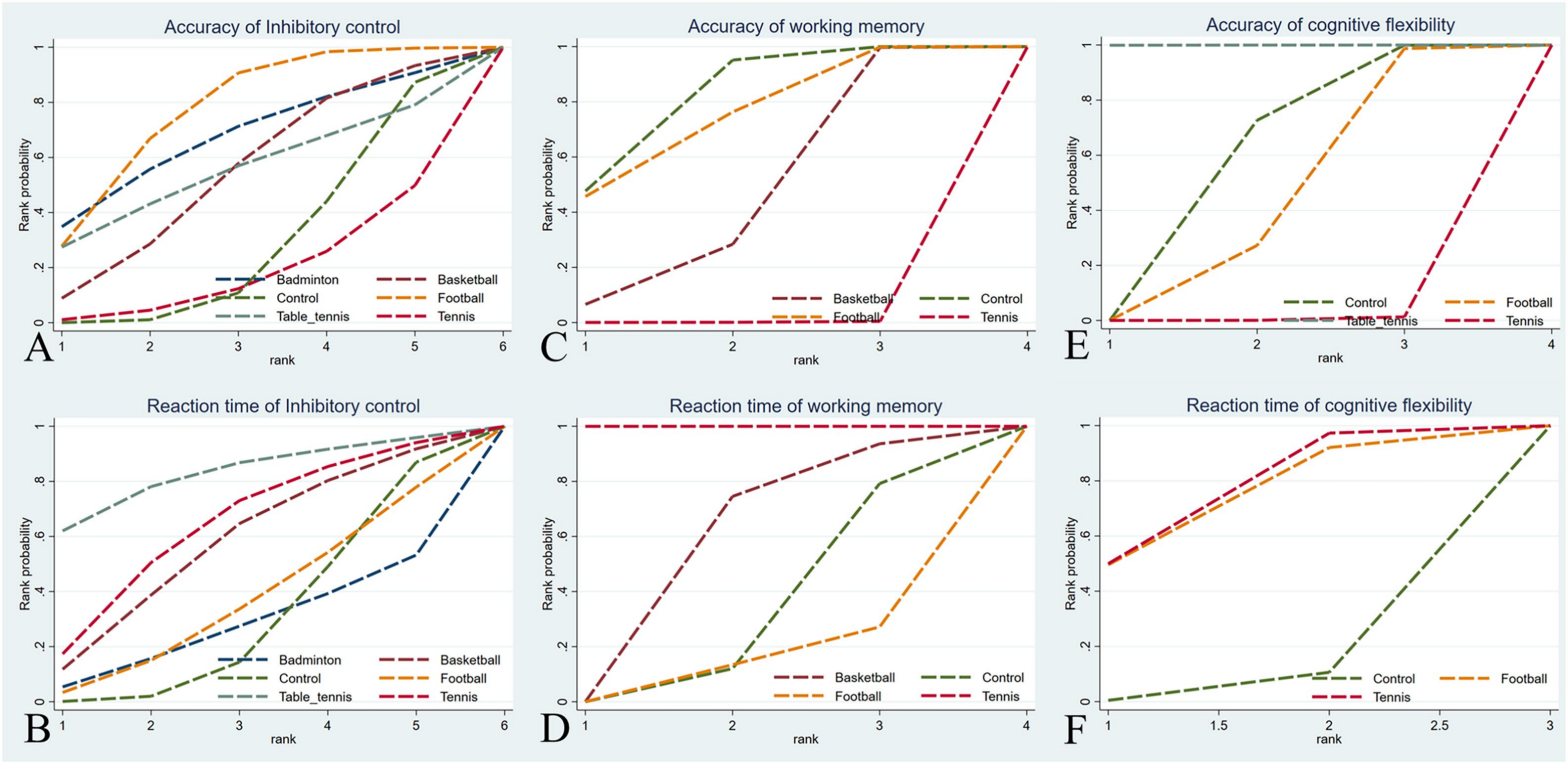
Cumulative ranking probability graph. The surface under the cumulative ranking curve (SUCRA) value is the probability each treatment has of being among the best of those in the network, with larger values representing higher ranking probabilities. **(A)** Accuracy of inhibitory control, **(B)** reaction time of Inhibitory control, **(C)** accuracy of working memory, **(D)** reaction time of working memory, **(E)** accuracy of cognitive flexibility, **(F)** reaction time of cognitive flexibility.

The accuracy of IC was reported in 10 studies involving 875 participants and five ball sports interventions: badminton, basketball, football, table tennis, and tennis. The results of the pairwise meta-analysis showed that ball sport interventions were associated with improvements in IC accuracy compared with the control group, with an overall *I^2^* value of 94.94% (Supplementary Table S4). The results of the NMA indicated that football showed a statistically significant advantage over the control group (SMD = 3.92, 95% CI: 0.44, 7.26). No statistically significant differences were observed in pairwise comparisons between other interventions (Supplementary Table S5). The potential ranking order of effectiveness for improving accuracy of IC across different interventions was as follows: football (SUCRA = 76.69%), badminton (SUCRA = 66.95%), and table tennis (SUCRA = 54.92%) (Figure 3A).

The reaction time of IC was reported in 8 studies involving 703 participants and five ball sports interventions: badminton, basketball, football, table tennis, and tennis. Pairwise meta-analysis did not indicate a statistically significant association between ball sport interventions and improvements in IC reaction time, with an overall *I^2^* value of 94.13% (Supplementary Table S4). The results of the NMA indicated that no statistically significant differences were found in pairwise comparisons between the interventions (Supplementary Table S5). The potential ranking order of effectiveness for improving reaction time of IC across different interventions was as follows: table tennis (SUCRA = 82.91%), tennis (SUCRA = 64.08%), and basketball (SUCRA = 57.46%) (Figure 3B).

#### 3.4.2 Working memory performance

The accuracy of WM was reported in 5 studies involving 370 participants and three ball sports interventions: basketball, football, and tennis. The results of the pairwise meta-analysis showed that a statistically significant association between the control group and improvements in WM accuracy compared to ball sport interventions, with an overall *I^2^* value of 97.93% (Supplementary Table S4). The results of the NMA indicated that compared to tennis, control group (SMD = 7.29, 95% CI: 3.78, 10.78), basketball (SMD = 6.21, 95% CI: 2.27, 10.16), and football (SMD = 7.20, 95% CI: 2.90, 11.52) each showed a statistically significant advantage over tennis in improving WM accuracy among children and adolescents. No statistically significant differences were observed in pairwise comparisons between other interventions (Supplementary Table S6). The potential ranking order of effectiveness for improving accuracy of WM across different interventions was as follows: control (SUCRA = 80.93%), football (SUCRA = 73.96%), and basketball (SUCRA = 44.91%) (Figure 3C).

The reaction time of WM was reported in 5 studies involving 370 participants and three ball sports interventions: basketball, football, and tennis. The results of the pairwise meta-analysis showed that ball sport interventions were associated with improvements in WM reaction time compared with the control group, with an overall *I^2^* value of 89.67% (Supplementary Table S4). NMA analysis showed that tennis showed a statistically significant advantage over basketball (SMD = −170.41, 95% CI: −234.09, −105.79), football (SMD = −195.22, 95% CI: −267.73, −121.00), and the control group (SMD = −180.87, 95% CI: −240.08, −121.47). No statistically significant differences were observed in pairwise comparisons between other interventions (Supplementary Table S6). The potential ranking order of effectiveness for improving reaction time of WM across different interventions was as follows: tennis (SUCRA = 99.99%), basketball (SUCRA = 56.06%), and control (SUCRA = 30.41%) (Figure 3D).

#### 3.4.3 Cognitive flexibility performance

The accuracy of CF was reported in 4 studies involving 205 participants and three ball sports interventions: football, table tennis, and tennis. Pairwise meta-analysis did not indicate a statistically significant association between ball sport interventions and improvements in CF accuracy, with an overall *I^2^* value of 0% (Supplementary Table S4). The NMA analysis revealed that table tennis showed a statistically significant advantage over football (SMD = 8.08, 95% CI: 3.72, 12.35) and the control group (SMD = 7.51, 95% CI: 3.69, 11.23). Compared to tennis, the control (SMD = 3.47, 95% CI: 2.35, 4.46), football (SMD = 2.88, 95% CI: 0.52, 5.34), and table tennis (SMD = 10.99, 95% CI: 6.99, 14.87) showed a statistically significant advantage in the accuracy of CF. No statistically significant differences were observed in pairwise comparisons between other interventions (Supplementary Table S7). The potential ranking order of effectiveness for improving accuracy of CF across different interventions was as follows: table tennis (SUCRA = 99.97%), control (SUCRA = 57.33%), and football (SUCRA = 42.28%) (Figure 3E).

The reaction time of CF was reported in 2 studies involving 151 participants and two ball sports interventions: football and tennis. The results of the pairwise meta-analysis showed that ball sport interventions were associated with improvements in CF reaction time, with an overall *I^2^* value of 88.32% (Supplementary Table S4). However, the NMA indicated that no statistically significant differences were found in pairwise comparisons between the interventions (Supplementary Table S7). The potential ranking order of effectiveness for improving the reaction time of CF across different interventions was as follows: tennis (SUCRA = 73.63%), football (SUCRA = 70.82%), and control (SUCRA = 57.33%) (Figure 3F).

#### 3.4.4 Results of subgroup and sensitivity analyses

To explain the high statistical heterogeneity observed across outcomes, subgroup analyses were conducted. For IC and WM accuracy and reaction time, country, population type, age, exercise modality, and session duration were included as moderator variables. The results showed that population type could partially explain the heterogeneity observed in IC accuracy (typically developing: SMD = −0.06, 95% CI: −0.17, 0.06, I^2^ = 72.2%; clinical: SMD = 1.09, 95% CI: 0.88, 1.30, I^2^ = 62.8%). For IC reaction time, single-session exercise duration may have moderated the direction of the effect (≤30 min: SMD = −0.28, 95% CI: −0.70, 0.13, I^2^ = 62.1%; 31–60 min: SMD = −0.33, 95% CI: −0.72, 0.07, I^2^ = 77.5%; >60 min: SMD = 0.30, 95% CI: −0.10, 0.72, I^2^ = 73.6%). For WM accuracy and reaction time, the majority of subgroups showed I^2^ values greater than 90%. Across the four outcomes, substantial differences in effect direction across countries suggest that country may be an important source of heterogeneity; extremely high heterogeneity within the same age groups (I^2^ ranging from 85.0 to 96.6%) indicates that age alone cannot effectively explain the observed variability. Similarly, the heterogeneity within the exercise modality subgroups was also high (I^2^ ranging from 76.9 to 94.3%), suggesting that exercise modality could not account for the observed variability. The results of the subgroup analyses are presented in Supplementary Table S8.

Due to the sparsity of the network structure, a leave-one-out sensitivity analysis was conducted only for NMA outcomes with four or more included studies. After sequentially excluding each study, the effect sizes for IC accuracy, IC reaction time, WM accuracy, WM reaction time, and CF accuracy all remained within their original 95% confidence intervals, with no change in direction (Supplementary Figures S3A–E).

To exclude the confounding influence of clinical populations, the analysis was restricted to typically developing children and adolescents. Separate NMAs were conducted for IC accuracy and reaction time in the typically developing sample, including 6 studies involving 4 types of ball sports (basketball, football, badminton, and tennis). Compared with the full-sample network, the network structures restricted to the typically developing sample changed consistently across both outcomes, with the direct comparison edge “table tennis vs. control” disappearing (Supplementary Figures S2A,B). Regarding accuracy, the SUCRA rankings showed marked changes: badminton increased from second place in the full sample (SUCRA = 66.95%) to first place in the analysis restricted to the typically developing sample (SUCRA = 91.91%), whereas football decreased from first to fourth place. Regarding reaction time, owing to the absence of table tennis, tennis rose from second place in the full sample (SUCRA = 64.08%) to first place in the analysis restricted to the typically developing sample (SUCRA = 70.16%) (Supplementary Table S9). Separate NMAs restricted to the typically developing sample were performed for WM accuracy and reaction time, including 4 studies involving 3 ball sports (basketball, football, and tennis) (Supplementary Figure S2C,D). Compared with the full-sample network, the network structure restricted to the typically developing sample remained unchanged, and the SUCRA rankings of the interventions for both accuracy and reaction time were consistent with those from the full-sample analysis (Supplementary Table S9). For CF accuracy, an NMA restricted to the typically developing sample included 2 studies involving 2 ball sports (football and tennis) (Supplementary Figure S2E). Owing to the absence of table tennis, the control group rose from second position in the full sample (SUCRA = 57.33%) to first position in the analysis restricted to the typically developing sample (SUCRA = 86.42%) (Supplementary Table S9). Supplementary Tables S3–S7 report the re-analysis results for the typically developing sample. These tables present the global inconsistency and heterogeneity, pairwise meta-analysis, and network meta-analysis matrix for each outcome.

#### 3.4.5 Publication bias and small sample effect test

**Figure 4:**
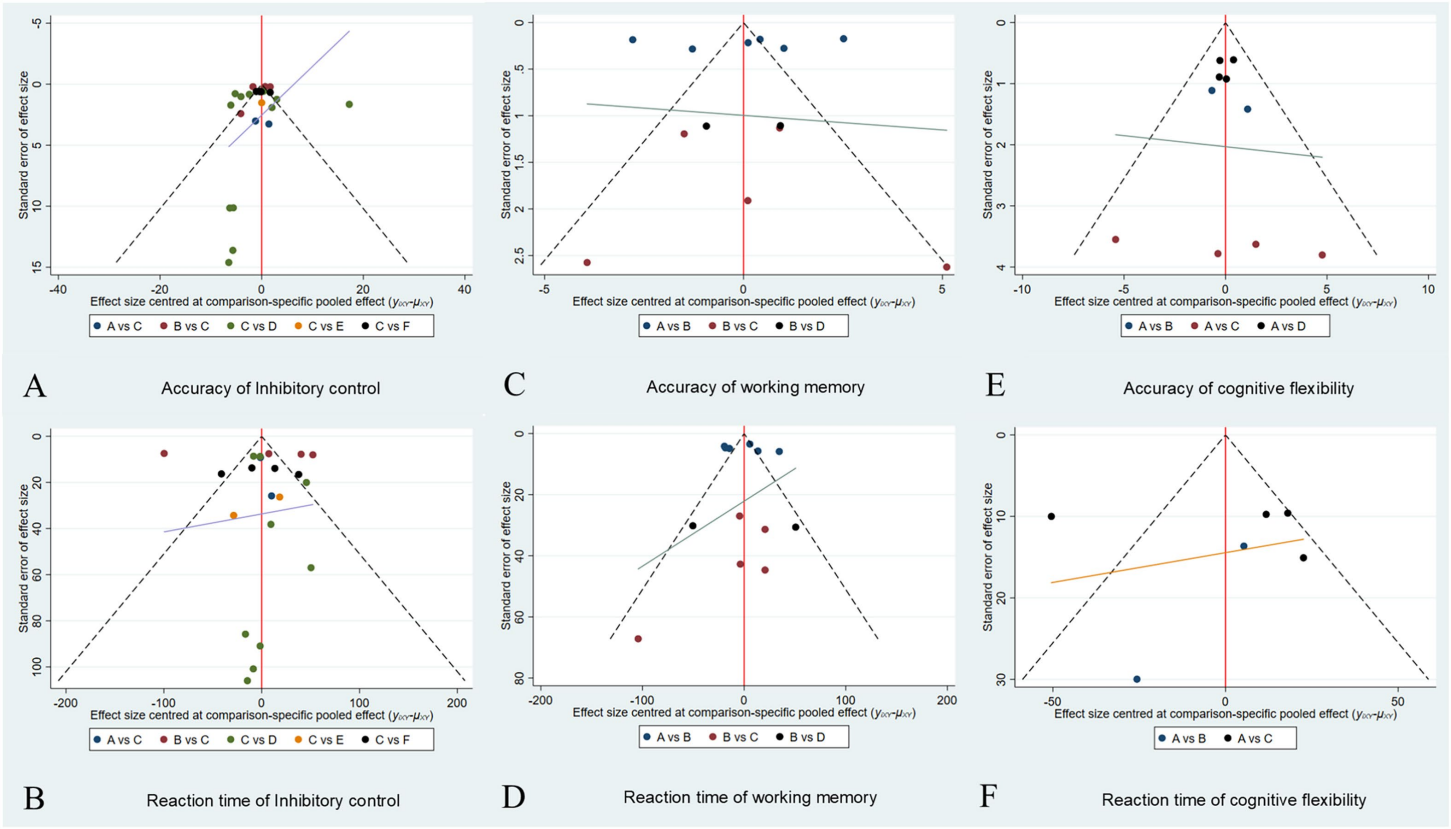
Network meta-analysis funnel graph. Publication bias or small sample effect test. **(A)** Accuracy of inhibitory control, **(B)** reaction time of inhibitory control, **(C)** accuracy of working memory, **(D)** reaction time of working memory, **(E)** accuracy of cognitive flexibility, **(F)** reaction time of cognitive flexibility.

For the accuracy and reaction time of WM, as the funnel plot was visibly slanted and the *p*-value obtained from Egger’s test was lower than 0.05, it indicated the presence of publication bias. To further investigate the potential impact of selective reporting, a trim-and-fill analysis was conducted. The assessment suggested that publication bias had a limited influence on the pooled estimates. In contrast, regarding the IC performance and CF performance indicators, the funnel plots were basically symmetrical. Moreover, when using Egger’s test to assess publication bias for IC performance and CF performance indicators, the results suggested that the *p*-values were greater than 0.05, indicating that the data for these aspects are relatively more reliable and less likely to be affected by potential biases in the publication process (Figure 4, Supplementary Table S10).

## 4 Discussion

This systematic review and NMA explored the impact of ball sports on each subdomain of the EF in children and adolescents, focusing on three core subdomains, including IC, WM, and CF. The research results indicate that the existing evidence is insufficient to support the conclusion that any ball game consistently outperforms the control group across all EF outcomes. The effects of ball games on EF subdomains are heterogeneous. SUCRA rankings suggest that certain ball games may have potential advantages for specific components of executive function, but some network comparisons have not reached statistical significance. Therefore, the results of this study should be interpreted as exploratory and hypothesis-generating evidence.

### 4.1 Inhibitory control

Ball sports may have potential advantages in improving IC accuracy in children and adolescents, with the SUCRA rankings suggesting that football may demonstrate relatively greater effectiveness. Regarding IC reaction time, although the network comparisons did not reveal statistically significant differences, the SUCRA rankings suggested that table tennis may have a potential advantage. Different ball sports may impose distinct attentional demands, leading to these differences. At the cognitive level, the potential advantage of football may stem from its continuous demands on selective attention, sustained attention, and interference inhibition. In dynamic multi-agent environments, players are required to continuously extract task-relevant information from complex environmental stimuli while suppressing dominant responses that conflict with current rules or tactical objectives (Habekost et al., 2024; Heilmann et al., 2024; Huijgen et al., 2015). Such repeated engagement in attentional regulation and response inhibition may provide sustained training stimuli for improving IC accuracy. In contrast, table tennis relies more heavily on rapid attentional shifting, phasic alertness, and visuomotor integration. Players must rapidly reorient attention toward a fast-moving ball within an extremely short time window and quickly prepare and execute motor responses. Repeated activation of rapid attentional reallocation and high-speed information processing may preferentially facilitate processing speed under IC conditions (Huang et al., 2024; Ren et al., 2025). Because different ball sports vary in environmental dynamism, informational complexity, and attentional regulation demands, they may exert differential influences on specific executive function subdomains. Neuroimaging evidence partially supports these cognitive interpretations. Functional near-infrared spectroscopy studies have shown that, following football participation, brain activity is mainly concentrated in the temporal lobe, frontal lobe, and cerebellum, particularly in the posterior superior temporal gyrus (pSTG) and the temporo-occipital middle temporal gyrus (toMTG) (Dong et al., 2024). In addition, functional connectivity studies have demonstrated that table tennis athletes exhibit increased dynamic functional connectivity (dFC) from the left frontotemporal region to the prefrontal cortex, primarily involving the left middle frontal gyrus, the left superior frontal gyrus (medial), and the left superior frontal gyrus (dorsolateral) (Li et al., 2023). These brain regions play critical roles in visual processing, perceptual integration, spatial processing, and functional brain connectivity (Jo et al., 2019).

### 4.2 Working memory

The effects of different ball sports on WM outcomes exhibited clear specificity. In terms of reaction time, ball sports overall were associated with faster WM responses, with tennis ranking highest in the SUCRA analysis. Tennis may be associated with faster responses on WM tasks. Nonetheless, regarding the accuracy of WM, no significant advantage of ball sports was detected; the control condition achieved a relatively high ranking, whereas tennis received a lower ranking. These patterns could be tentatively explained by the rapid perception–decision–action demands emphasized in tennis may preferentially benefit processing speed under high cognitive load rather than the precise maintenance and manipulation of information. This interpretation is consistent with empirical evidence. Xu et al. reported that, compared with children who underwent short-term tennis training, children aged 8–12 years who received long-term tennis training demonstrated significantly shorter reaction times in WM tasks, with no significant differences in accuracy (Zagatto et al., 2018). From the perspective of sport-related attentional demands, tennis is characterized by fast pace, frequent changes, and high uncertainty in ball trajectory, speed, and landing location, requiring participants to continuously process visual information and make motor decisions within very short time windows (Yufei and Ningning, 2026). This process imposes heavy demands on rapid attentional shifting and sustained attention. Such transient information processing under high cognitive load could potentially influence WM reaction time. However, it is important to note that Egger’s test detected the presence of publication bias, the results of the trim-and-fill analysis indicated that the extent of publication bias was limited and the study conclusions were relatively robust, the possibility of overestimated intervention effects cannot be excluded. Therefore, all WM findings should be interpreted with considerable caution.

### 4.3 Cognitive flexibility

The effects of different ball sports on CF differed between accuracy and reaction time outcomes. NMA showed that table tennis ranked highest in SUCRA for accuracy of CF, while tennis ranked highest for reaction time of CF. This suggests that racket sports may show relatively higher ranking probabilities for certain outcomes, although this does not constitute definitive evidence of superiority. Table tennis and tennis are typically associated with high frequency contextual changes and immediate decision-making demands, which may theoretically facilitate CF processes (Álvarez-Bueno et al., 2017). However, the current NMA does not provide sufficient evidence to support the conclusion that racket sports are overall superior to other ball sports. Specifically, for accuracy outcomes, no statistically significant differences were observed between overall ball sports interventions and control conditions. Network comparisons indicated statistically significant differences between table tennis, football, and the control group compared with tennis, but these differences primarily reflect the direction of relative effects between interventions rather than indicating a detrimental effect of tennis. For reaction time outcomes, ball sports interventions overall tended to shorten response times, yet no statistically significant differences were found between different ball sports or compared with control conditions in the NMA. This discrepancy reflects the distinction between pairwise meta-analyses, which emphasize average intervention effects, and NMA, which focuses on relative differences between specific interventions. Given the limited available evidence, network model estimates may be influenced by insufficient sample sizes and uncertainty in indirect comparisons, precluding firm conclusions regarding the superiority of any single ball sport in reaction time outcomes. Therefore, the observed SUCRA rankings should not be interpreted as definitive evidence of superiority.

### 4.4 Interpretation of subgroup and sensitivity analyses findings

The subgroup analyses provided additional insights into potential sources of heterogeneity. Population type appeared to partially explain the heterogeneity observed in IC accuracy outcomes. For IC reaction time, single-session exercise duration may have moderated the direction of the effect. Furthermore, substantial differences in effect direction across countries suggest that country may be an important source of heterogeneity. The high heterogeneity observed in the current evidence base is likely the result of multiple interacting factors. Among these, population type and country differences may have played a relatively important role, whereas age, exercise modality, and session duration failed to substantially reduce within-subgroup heterogeneity. This conclusion is constrained by the generally small number of studies within each subgroup (mostly n ≤ 4). Future studies should accumulate more primary research that adopts standardized task paradigms, consistent measurement time points, and detailed reporting of intervention protocols to systematically assess the specific sources of heterogeneity through methods such as meta-regression.

The sensitivity analyses further demonstrated that the NMA findings were highly dependent on sample composition. After restricting the analyses to typically developing children and adolescents, substantial changes were observed in the SUCRA rankings for IC outcomes (both accuracy and reaction time), as well as for CF accuracy. These findings suggest that the apparent superiority of certain interventions in the full-sample analyses may partly reflect the inclusion of clinical populations rather than stable sport-specific effects. In contrast, the SUCRA rankings for WM outcomes remained relatively stable after restricting analyses to typically developing samples, suggesting comparatively greater robustness for WM-related findings. Due to the extremely limited number of studies, CF reaction time could not be assessed.

### 4.5 Functional roles of accuracy and reaction time in real-world contexts

This study employed both accuracy and reaction time as dual indicators to assess performance across subdomains of EF. Accuracy and reaction time reflect two relatively independent yet complementary dimensions of cognitive processing. Accuracy primarily measures the correctness of an individual’s responses during task execution, capturing the precision of cognitive control and error monitoring; reaction time, in contrast, reflects the speed and efficiency of information processing (Duggins and Eliasmith, 2025). Accuracy is particularly critical in contexts where errors carry high costs and cautious decision-making is required, whereas reaction time assumes greater importance in time-pressured situations demanding rapid responses. The distinct attentional demands cultivated by different ball sports may map onto these two dimensions and extend to real-world learning, daily life, and decision-making behaviors. For example, football requires participants to continuously suppress actions that are incompatible with rules or tactical goals in a dynamically changing environment (Bari and Robbins, 2013; Huijgen et al., 2015). Such high demands on interference suppression may preferentially contribute to improvements in IC accuracy, thereby benefiting everyday situations that require sustained focus and resistance to irrelevant distractions. Tennis emphasizes perceptual judgment and motor selection in response to rapid incoming balls within extremely short time windows, which may enhance WM reaction time by accelerating information updating (Xu et al., 2022). This may hold relevance for scenarios that require rapid recall of previously acquired information and immediate judgment, such as classroom responses or urgent decision-making. Table tennis is characterized by high-frequency contextual changes and instantaneous response demands, potentially promoting CF accuracy and reflecting the ability to flexibly adjust responses in the face of unpredictable changes—an ability that is particularly important for adapting to novel environments and multitasking (Ren et al., 2025; Xu et al., 2022).

Across the subdomains of EF, different ball sports exhibited certain differential effects; however, the current evidence is insufficient to support definitive conclusions regarding superiority. Future studies should employ larger sample sizes and direct head-to-head RCTs to verify the specific effects of different ball sports on EF subdomains.

## 5 Limitations

There are several limitations to this study that warrant consideration. First, the number of studies specifically examining the effects of ball sports on EF in children and adolescents is relatively limited, and direct head-to-head comparisons between different types of ball sports are scarce. As a result, some comparisons within the network relied primarily on indirect evidence. Under conditions of small sample sizes, the precision of network meta-analytic estimates may be reduced, leading to wider confidence intervals and affecting the stability of effect estimates as well as the reliability of ranking results. Consequently, the observed SUCRA rankings should not be interpreted as definitive evidence of superiority. In addition, several outcomes were informed by only two to five studies, further limiting the robustness of the findings. Second, different studies employed various EF assessment tools, making the results susceptible to methodological heterogeneity. Although accuracy and reaction time were analyzed separately in the present study to reduce bias arising from mixed indicators, differences in task paradigms may still influence the interpretation of findings. Third, heterogeneity remained high within most subgroups, indicating that the observed variability likely arose from multiple interacting factors, including differences in participant characteristics, intervention protocols, exercise duration and intensity, cognitive engagement, and outcome assessment methods. Because the number of included studies within most subgroups was very small, the subgroup findings should be considered exploratory rather than confirmatory. Fourth, all included studies were RCTs; however, given the nature of exercise interventions, blinding is inherently difficult to implement. Therefore, the possibility of publication bias cannot be completely excluded. Fifth, publication bias and small-study effects were detected for both accuracy and reaction time outcomes of WM. Although trim-and-fill analyses suggested that the influence of publication bias on the pooled estimates was limited, the possibility of inflated effect sizes cannot be excluded. Furthermore, most studies were conducted in China, while evidence from other countries and cultural contexts was relatively limited. Taken together, the findings of this study should be considered a synthesis of the currently available evidence rather than definitive conclusions. Future research should employ rigorously designed RCTs with larger sample sizes and direct comparisons between different ball sports, as well as standardized EF assessment paradigms, to improve the quality of evidence and clarify the relative effects of specific ball sports.

## 6 Conclusion

This NMA indicates that ball sports have subdomain-specific effects on EF in children and adolescents, with no single sport consistently superior across all outcomes. Football showed the highest probability of improving the accuracy of IC; tennis was most likely to benefit the reaction time of WM; and table tennis ranked highest for the accuracy of CF. However, sensitivity analyses restricted to typically developing children and adolescents revealed notable changes in network structures and SUCRA rankings for IC accuracy, IC reaction time, and CF accuracy, indicating that certain findings from the full-sample analysis may not be directly generalizable to typically developing populations. In addition, many comparisons were not statistically significant, and confidence in the evidence was limited by high heterogeneity, small sample sizes, and potential publication bias. Overall, these findings should be considered exploratory. Future well designed RCTs with standardized protocols and comprehensive EF assessments are needed to determine whether specific ball sports can be targeted to enhance particular subdomains of EF.

## Data Availability

The original contributions presented in the study are included in the article/Supplementary material, further inquiries can be directed to the corresponding author/s.
