# Supplementary material for: Comparative effects of ball sports on executive function subdomains in children and adolescents: a network meta-analysis
Source: Front Psychol. 2026 Jun 10;17:1830417. doi: 10.3389/fpsyg.2026.1830417 (PMC13290521; doi:10.3389/fpsyg.2026.1830417)

**Supplement Material**

**Effects of Ball Sports Interventions on Executive Function Subdomains in Children and Adolescents: A Network Meta-Analysis**

Table S1 Web of Science search strategy.**2**

Table S2 CINeMA Results.**2**

Table S3 Global inconsistency and heterogeneity of each outcome...**5**

Table S4 Pairwise meta-analysis.**6**

Table S5 Matrix of the network meta-analysis results for inhibitory control performance.**8**

Table S6 Matrix of the network meta-analysis results for working memory performance.**9**

Table S7 Matrix of the network meta-analysis results for cognitive flexibility performance.**10**

Table S8 Subgroup Analysis.**10**

Table S9 Ranking of SUCRA (Surface Under the Cumulative Ranking Curve) values for typically developing children and adolescents.**13**

Table S10 Egger’s publication test.**14**

Figure S1 Risk of bias graph.**15**

Figure S2 Network graph for typically developing children and adolescents.**16**

Figure S3 Leave‑one‑out sensitivity analysis results graph.**17**

Supplementary Material

# **Table S1 Web of Science search strategy.**

| Search Query | Results | Date Run |
| --- | --- | --- |
| 1: (TS=(ball) OR TS=(ball games) OR TS=(ball sports) OR TS=(Basketball) OR TS=(Football) OR TS=(Soccer) OR TS=(Tennis) OR TS=(Badminton) OR TS=(Volleyball) OR TS=(Hockey) OR TS=(Softball) OR TS=(Handball) OR TS=(table tennis) OR TS=(ping pong) OR TS=(ice hockey) OR TS=(rugby)) NOT (SILOID==("PPRN")) and Preprint Citation Index (Exclude – Database) | 224925 | Fri Nov 29 2025 18:56:09 GMT+0800 |
| 2: (TS=(Adolescent) OR TS=(Adolescents) OR TS=(Adolescence) OR TS=(Female Adolescent) OR TS=(Female Adolescents) OR TS=(Male Adolescent) OR TS=(Male Adolescents) OR TS=(Youth) OR TS=(Youths) OR TS=(Teens) OR TS=(Teen) OR TS=(Teenagers) OR TS=(Teenager) OR TS=(Students) OR TS=(Student) OR TS=(Child) OR TS=(Children)) NOT (SILOID==("PPRN")) and Preprint Citation Index (Exclude – Database) | 6474035 | Fri Nov 29 2025 18:56:20 GMT+0800 |
| 3: (TS=(Executive Function) OR TS=(Executive Functions) OR TS=(Executive Control) OR TS=(Executive Controls) OR TS=(executive dysfunction) OR TS=(cognitive control) OR TS=(Cognitions) OR TS=(Cognitive Function) OR TS=(Cognitive Functions) OR TS=(inhibitory control) OR TS=(Impulse-Control) OR TS=(impulse control) OR TS=(Reactive Inhibitions) OR TS=(response inhibition) OR TS=(inhibit) OR TS=(Working Memory) OR TS=(Working Memories) OR TS=(Shortterm Memories) OR TS=(Shortterm Memory) OR TS=(Cognitive Flexibility) OR TS=(Cognitive Flexibilities) OR TS=(Cognitive Inflexibility) OR TS=(Cognitive Inflexibilities) OR TS=(mental flexibility) OR TS=(problem-solving) OR TS=(decision making) OR TS=(problem solving)) NOT (SILOID==("PPRN")) and Preprint Citation Index (Exclude – Database) | 4900236 | Fri Nov 29 2025 18:56:29 GMT+0800 |
| 4: #1 AND #2 AND #3 and Preprint Citation Index (Exclude – Database) | 3459 | Fri Nov 29 2025 18:56:37 GMT+0800 |

# **Table S2 CINeMA Results**

| **Comparison** | **Number of studies** | **Within-study bias** | **Reporting bias** | **Indirectness** | **Imprecision** | **Heterogeneity** | **Incoherence** | **Confidence rating** |
| --- | --- | --- | --- | --- | --- | --- | --- | --- |
| Accuracy performance of inhibitory control | | | | | | | | |
| Badminton:Control | 2 | Some concerns | Low risk | No concerns | Major concerns | No concerns | Major concerns | Very low |
| Basketball:Control | 5 | Some concerns | Low risk | No concerns | Some concerns | Some concerns | Major concerns | Low |
| Control:Football | 13 | Some concerns | Low risk | No concerns | No concerns | Some concerns | Major concerns | Low |
| Control:Table_tennis | 1 | Some concerns | Low risk | No concerns | Major concerns | No concerns | Major concerns | Very low |
| Control:Tennis | 4 | Major concerns | Low risk | No concerns | Some concerns | Some concerns | Major concerns | Very low |
| Badminton:Basketball | 0 | Some concerns | Low risk | No concerns | Major concerns | No concerns | Major concerns | Very low |
| Badminton:Football | 0 | Some concerns | Low risk | No concerns | Major concerns | No concerns | Major concerns | Very low |
| Badminton:Table_tennis | 0 | Some concerns | Low risk | No concerns | Major concerns | No concerns | Major concerns | Very low |
| Badminton:Tennis | 0 | Major concerns | Low risk | No concerns | Some concerns | Some concerns | Major concerns | Very low |
| Basketball:Football | 0 | Some concerns | Low risk | No concerns | Some concerns | Some concerns | Major concerns | Low |
| Basketball:Table_tennis | 0 | Some concerns | Low risk | No concerns | Major concerns | No concerns | Major concerns | Very low |
| Basketball:Tennis | 0 | Some concerns | Low risk | No concerns | No concerns | Major concerns | Major concerns | Very low |
| Football:Table_tennis | 0 | Some concerns | Low risk | No concerns | Major concerns | No concerns | Major concerns | Very low |
| Football:Tennis | 0 | Some concerns | Low risk | No concerns | No concerns | No concerns | Major concerns | Low |
| Table_tennis:Tennis | 0 | Major concerns | Low risk | No concerns | Major concerns | No concerns | Major concerns | Very low |
| Reaction time performance of inhibitory control | | | | | | | | |
| Badminton:Control | 2 | Some concerns | Low risk | No concerns | Major concerns | No concerns | Major concerns | Very low |
| Basketball:Control | 4 | Some concerns | Low risk | No concerns | Major concerns | No concerns | Major concerns | Very low |
| Control:Football | 9 | No concerns | Low risk | No concerns | Major concerns | No concerns | Major concerns | Very low |
| Control:Table_tennis | 2 | Some concerns | Low risk | No concerns | Major concerns | No concerns | Major concerns | Very low |
| Control:Tennis | 4 | Major concerns | Low risk | No concerns | Some concerns | Some concerns | Major concerns | Very low |
| Badminton:Basketball | 0 | Some concerns | Low risk | No concerns | Major concerns | No concerns | Major concerns | Very low |
| Badminton:Football | 0 | Some concerns | Low risk | No concerns | Major concerns | No concerns | Major concerns | Very low |
| Badminton:Table_tennis | 0 | Some concerns | Low risk | No concerns | Major concerns | No concerns | Major concerns | Very low |
| Badminton:Tennis | 0 | Major concerns | Low risk | No concerns | Major concerns | No concerns | Major concerns | Very low |
| Basketball:Football | 0 | Some concerns | Low risk | No concerns | Major concerns | No concerns | Major concerns | Very low |
| Basketball:Table_tennis | 0 | Some concerns | Low risk | No concerns | Major concerns | No concerns | Major concerns | Very low |
| Basketball:Tennis | 0 | Major concerns | Low risk | No concerns | Major concerns | No concerns | Major concerns | Very low |
| Football:Table_tennis | 0 | Some concerns | Low risk | No concerns | Major concerns | No concerns | Major concerns | Very low |
| Football:Tennis | 0 | Some concerns | Low risk | No concerns | Major concerns | No concerns | Major concerns | Very low |
| Table_tennis:Tennis | 0 | Major concerns | Low risk | No concerns | Major concerns | No concerns | Major concerns | Very low |
| Accuracy performance of working memory | | | | | | | | |
| Basketball:Control | 6 | No concerns | Low risk | No concerns | Some concerns | Some concerns | Major concerns | Very low |
| Control:Football | 5 | No concerns | Low risk | No concerns | Major concerns | No concerns | Major concerns | Very low |
| Control:Tennis | 2 | Major concerns | Low risk | No concerns | No concerns | No concerns | Major concerns | Very low |
| Basketball:Football | 0 | No concerns | Low risk | No concerns | Major concerns | No concerns | Major concerns | Very low |
| Basketball:Tennis | 0 | Some concerns | Low risk | No concerns | No concerns | No concerns | Major concerns | Low |
| Football:Tennis | 0 | Some concerns | Low risk | No concerns | No concerns | No concerns | Major concerns | Low |
| Reaction time performance of working memory | | | | | | | | |
| Basketball:Control | 6 | No concerns | Low risk | No concerns | No concerns | No concerns | Major concerns | Low |
| Control:Football | 5 | No concerns | Low risk | No concerns | No concerns | Some concerns | Major concerns | Low |
| Control:Tennis | 2 | Major concerns | Low risk | No concerns | No concerns | No concerns | Major concerns | Very low |
| Basketball:Football | 0 | No concerns | Low risk | No concerns | Some concerns | No concerns | Major concerns | Low |
| Basketball:Tennis | 0 | Some concerns | Low risk | No concerns | No concerns | No concerns | Major concerns | Low |
| Football:Tennis | 0 | Some concerns | Low risk | No concerns | No concerns | No concerns | Major concerns | Low |
| Accuracy performance of cognitive flexibility | | | | | | | | |
| Control:Football | 2 | Some concerns | Low risk | No concerns | Some concerns | No concerns | Some concerns | Low |
| Control:Table_tennis | 4 | Some concerns | Low risk | No concerns | No concerns | No concerns | Some concerns | Moderate |
| Control:Tennis | 4 | Major concerns | Low risk | No concerns | No concerns | No concerns | Some concerns | Moderate |
| Football:Table_tennis | 0 | Some concerns | Low risk | No concerns | No concerns | No concerns | Some concerns | Moderate |
| Football:Tennis | 0 | Major concerns | Low risk | No concerns | No concerns | No concerns | Some concerns | Low |
| Table_tennis:Tennis | 0 | Some concerns | Low risk | No concerns | No concerns | No concerns | Some concerns | Moderate |
| Reaction time performance of cognitive flexibility | | | | | | | | |
| Control:Football | 2 | Some concerns | Low risk | No concerns | No concerns | Some concerns | Major concerns | Low |
| Control:Tennis | 4 | Major concerns | Low risk | No concerns | No concerns | Some concerns | Major concerns | Very low |
| Football:Tennis | 0 | Major concerns | Low risk | No concerns | No concerns | Major concerns | Major concerns | Very low |

# **Table S****3** **Global inconsistency and heterogeneity of each outcome.**

| Outcomes | Consistency model | | | | | Inconsistency model | | | | | dDIC |
| --- | --- | --- | --- | --- | --- | --- | --- | --- | --- | --- | --- |
|  | Dbar | data points | pD | DIC | *I^2^* | Dbar | data points | pD | DIC | *I^2^* |  |
| Inhibitory Control performance | | | | | | | | | | | |
| Total | | | | | | | | | | | |
| Accuracy | 48.17 | 50 | 45.90 | 94.07 | 0% | 48.10 | 50 | 45.86 | 93.96 | 0% | 0.11 |
| Reaction Time | 37.88 | 42 | 36.87 | 74.75 | 0% | 37.88 | 42 | 36.88 | 74.77 | 0% | 0.02 |
| Typically developing | | | | | | | | | | | |
| Accuracy | 25.89 | 26 | 22.61 | 48.50 | 3% | 25.80 | 26 | 22.54 | 48.35 | 3% | 0.15 |
| Reaction Time | 23.29 | 26 | 22.44 | 45.74 | 0% | 23.26 | 26 | 22.41 | 45.67 | 0% | 0.07 |
| Working Memory performance | | | | | | | | | | | |
| Total | | | | | | | | | | | |
| Accuracy | 26.63 | 26 | 24.00 | 50.63 | 6% | 26.65 | 26 | 24.01 | 50.66 | 6% | 0.01 |
| Reaction Time | 26.73 | 26 | 22.72 | 49.45 | 6% | 26.71 | 26 | 22.75 | 49.47 | 6% | 0.02 |
| Typically developing | | | | | | | | | | | |
| Accuracy | 23.48 | 24 | 22.44 | 45.92 | 2% | 23.41 | 24 | 22.38 | 45.80 | 2% | 0.12 |
| Reaction Time | 23.80 | 24 | 21.51 | 45.31 | 3% | 23.79 | 24 | 21.51 | 45.30 | 3% | 0.01 |
| Cognitive Flexibility performance | | | | | | | | | | | |
| Total | | | | | | | | | | | |
| Accuracy | 19.10 | 20 | 14.52 | 33.62 | 0.5% | 19.07 | 20 | 14.53 | 33.60 | 0.4% | 0.02 |
| Reaction Time | 11.91 | 12 | 11.58 | 23.49 | 8% | 11.90 | 12 | 11.56 | 23.46 | 8% | 0.03 |
| Typically developing | | | | | | | | | | | |
| Accuracy | 10.21 | 12 | 9.31 | 19.52 | 0% | 10.21 | 12 | 9.29 | 19.50 | 0% | 0.02 |

# **Table S4 Pairwise meta-analysis**

| **Outcome indicator** | **Comparisons** | **Number of**  **study** | **SMD** | **95%CI** | **I² (%)** | **Global**  **I² (%)** |
| --- | --- | --- | --- | --- | --- | --- |
| Inhibitory Control performance | | | | | | |
| Total | | | | | | |
| Accuracy | Basketball:Control | 5 | 1.92 | -2.77, 6.71 | 97.86% | 94.94% |
|  | Football:Control | 13 | 3.92 | 0.44, 7.27 | 93.92% |  |
|  | Tennis:Control | 4 | -1.31 | -6.56, 3.93 | 69.48% |  |
|  | Badminton:Control | 2 | -3.63 | 4.86, 12.17 | 0.00% |  |
|  | Table_tennis:Control | 1 | 2.31 | -8.52, 13.12 | NA |  |
| Reaction Time | Basketball:Control | 4 | -17.66 | -65.10, 29.61 | 98.79% | 94.13% |
|  | Football:Control | 9 | -2.31 | -44.83, 40.43 | 22.49% |  |
|  | Tennis:Control | 4 | -23.09 | -71.78, 25.74 | 77.13% |  |
|  | Badminton:Control | 2 | 8.12 | -62.68, 78.87 | 0.00% |  |
|  | Table_tennis:Control | 2 | -50.61 | -128.81, 26.71 | 14.44% |  |
| Typically developing | | | | | | |
| Accuracy | Basketball:Control | 4 | 3.62 | -1.21, 8.47 | 97.86% | 84.80% |
|  | Football:Control | 8 | -0.31 | -2.16, 1.48 | 6.34% |  |
|  | Tennis:Control | 4 | -1.32 | -2.95, 0.32 | 70.10% |  |
|  | Badminton:Control | 2 | 3.62 | -1.21, 8.46 | 0.00% |  |
| Reaction Time | Basketball:Control | 4 | -17.61 | -66.96, 80.59 | 98.79% | 94.71% |
|  | Football:Control | 8 | -10.33 | -58.22, 38.86 | 0.00% |  |
|  | Tennis:Control | 4 | -23.14 | -73.60, 27.69 | 76.47% |  |
|  | Badminton:Control | 2 | 8.07 | -65.51, 80.59 | 0.00% |  |
| Working Memory performance | | | | | | |
| Total | | | | | | |
| Accuracy | Basketball:Control | 6 | -1.08 | -2.91, 0.74 | 98.94% | 97.93% |
|  | Football:Control | 5 | -0.11 | -2.61, 2.46 | 52.08% |  |
|  | Tennis:Control | 2 | -7.30 | -10.79, -3.82 | 25.05% |  |
| Reaction Time | Basketball:Control | 6 | -10.51 | -35.63, 14.54 | 94.33% | 89.67% |
|  | Football:Control | 5 | 14.52 | -30.11, 56.91 | 0.00% |  |
|  | Tennis:Control | 2 | -180.7 | -239.60, -120.91 | 81.83% |  |
| Typically developing | | | | | | |
| Accuracy | Basketball:Control | 6 | -1.08 | -2.84, 0.67 | 98.94% | 98.12% |
|  | Football:Control | 4 | -0.84 | -3.56, 1.74 | 29.75% |  |
|  | Tennis:Control | 2 | -7.30 | -10.69, -3.93 | 253.92 |  |
| Reaction Time | Basketball:Control | 6 | 10.42 | -13.97, 34.84 | 94.37% | 90.52% |
|  | Football:Control | 4 | 23.63 | -21.17, 68.89 | 0.00% |  |
|  | Tennis:Control | 2 | -181.39 | -239.77, -122.13 | 82.20% |  |
| Cognitive Flexibility performance | | | | | | |
| Total | | | | | | |
| Accuracy | Football:Control | 2 | -0.59 | -2.72, 1.57 | 0.00% | 0.00% |
|  | Tennis:Control | 4 | -3.49 | -4.67, -2.33 | 0.00% |  |
|  | Table_tennis:Control | 4 | -7.48 | 3.60, 11.41 | 25.56% |  |
| Reaction Time | Football:Control | 2 | -45.83 | -120.91, 25.72 | 14.26% | 88.32% |
|  | Tennis:Control | 4 | -46.49 | -94.81, 2.48 | 90.94% |  |
| Typically developing | | | | | | |
| Accuracy | Football:Control | 2 | -0.61 | -2.75, 1.62 | 0.00% | 0.00% |
|  | Tennis:Control | 4 | -3.48 | -4.70, -2.31 | 0.00% |  |

# **Table S5 Matrix of the network meta-analysis results for inhibitory control performance.**

| Accuracy | Badminton |  |  |  |  |  |
| --- | --- | --- | --- | --- | --- | --- |
|  | 1.72 (-8.04, 11.36) | Basketball |  |  |  |  |
|  | 3.65 (-4.89, 12.14) | 1.93 (-2.75, 6.7) | Control |  |  |  |
|  | -0.25 (-9.4, 8.91) | -2 (-7.72, 3.93) | -3.92 (-7.26, -0.44) * | Football |  |  |
|  | 1.32 (-12.37, 15.01) | -0.41 (-12.11, 11.45) | -2.34 (-13.13, 8.46) | 1.57 (-9.77, 12.84) | Table tennis |  |
|  | 4.98 (-5.02, 14.94) | 3.24 (-3.77, 10.35) | 1.32 (-3.91, 6.56) | 5.23 (-1.05, 11.44) | 3.65 (-8.3, 15.67) | Tennis |
| Reaction Time | Badminton |  |  |  |  |  |
|  | 25.79 (-58.55, 110.11) | Basketball |  |  |  |  |
|  | 8.15 (-61.92, 78.41) | -17.59 (-64.75, 29.64) | Control |  |  |  |
|  | 10.54 (-72.17, 92.21) | -15.28 (-79.21, 48.19) | 2.3 (-40.69, 44.9) | Football |  |  |
|  | 58.77 (-45.27, 163.73) | 32.99 (-57.23, 124.14) | 50.61 (-26.49, 128.83) | 48.33 (-39.79, 137.74) | Table tennis |  |
|  | 31.62 (-54.2, 116.69) | 5.71 (-62.36, 73.72) | 23.31 (-25.64, 72.1) | 21.06 (-43.49, 86.26) | -27.24 (-119.65, 63.72) | Tennis |
| Typically developing |  |  |  |  |  |  |
| Accuracy | Badminton |  |  |  |  |  |
|  | 3.59 (-3.13, 10.31) | Basketball |  |  |  |  |
|  | 3.59 (-1.99, 9.18) | 0.00 (-3.88, 3.89) | Control |  |  |  |
|  | 4.00 (-2.09, 10.32) | 0.36 (-4.22, 5.35) | 0.38 (-2.24, 3.36) | Football |  |  |
|  | 5.45 (-1.28, 12.16) | 1.87 (-3.68, 7.38) | 1.86 (-2.07, 5.79) | 1.5 (-3.53, 6.10) | Tennis |  |
| Reaction Time | Badminton |  |  |  |  |  |
|  | -9.88 (-88.04, 67.29) | Basketball |  |  |  |  |
|  | 8.46 (-49.27, 65.16) | 18.43 (-34.64, 71.18) | Control |  |  |  |
|  | 10.39 (-59.48, 77.44) | 20.33 (-45.73, 83.77) | 1.83 (-36.44, 38.31) | Football |  |  |
|  | 35.54 (-44.94, 115.19) | 45.47 (-31.35, 121.84) | 27.23 (-28.46, 82.67) | 25.18 (-41, 92.93) | Tennis |  |

# **Table S6** **Matrix of the network meta-analysis results for working memory performance.**

| Accuracy | Basketball |  |  |  |
| --- | --- | --- | --- | --- |
|  | -1.09 (-2.91, 0.75) | Control |  |  |
|  | -0.99 (-4.15, 2.1) | 0.09 (-2.45, 2.62) | Football |  |
|  | 6.21 (2.27, 10.16) * | 7.29 (3.78, 10.78) * | 7.2 (2.9, 11.52) * | Tennis |
| Reaction Time | Basketball |  |  |  |
|  | -10.48 (-35.51, 14.41) | Control |  |  |
|  | -24.79 (-73.9, 26.09) | -14.29 (-56.9, 29.93) | Football |  |
|  | 170.41 (105.79, 234.09) * | 180.87 (121.47, 240.08) * | 195.22 (121, 267.73) * | Tennis |
| Typically developing |  |  |  |  |
| Accuracy | Basketball |  |  |  |
|  | -1.09 (-2.83, 0.64) | Control |  |  |
|  | -0.23 (-3.33, 2.97) | 0.86 (-1.73, 3.54) | Football |  |
|  | 6.21 (2.46, 9.97) * | 7.29 (3.97, 10.65) * | 6.44 (2.16, 10.63) * | Tennis |
| Reaction Time | Basketball |  |  |  |
|  | -10.46 (-34.86, 13.74) | Control |  |  |
|  | -34.19 (-85.6, 16.61) | -23.73 (-68.91, 21.01) | Football |  |
|  | 170.61 (106.92, 234.52) * | 181.04 (122.22, 240.18) * | 204.77 (131.12, 278.94) * | Tennis |

# **Table S7 Matrix of the network meta-analysis results for cognitive flexibility performance.**

| Accuracy | Control |  |  |  |
| --- | --- | --- | --- | --- |
|  | 0.59 (-1.57, 2.7) | Football |  |  |
|  | -7.51 (-11.23, -3.69) * | -8.08 (-12.35, -3.72) * | Table tennis |  |
|  | 3.47 (2.35, 4.64) * | 2.88 (0.52, 5.34) * | 10.99 (6.99, 14.87) * | Tennis |
| Reaction Time | Control |  |  |  |
|  | 46.03 (-24.61, 121.47) | Football |  |  |
|  | 46.27 (-2.88, 94.41) | 0.24 (-89.87, 85.48) | Tennis |  |
| Typically developing |  |  |  |  |
| Accuracy | Control |  |  |  |
|  | 0.62 (-1.52, 2.66) | Football |  |  |
|  | 3.47 (2.35, 4.61) * | 2.86 (0.54, 5.3) * | Tennis |  |

# **Table S8 Subgroup Analysis.**

| Subgroup | | Number of Studies | Z-value | P-value | I² (%) | P-value | SMD (95% CI) |
| --- | --- | --- | --- | --- | --- | --- | --- |
| Inhibitory Control performance (Accuracy) | | | | | | | |
| Country | China | 4 | 4.35 | 0.000 | 62.8 | 0.045 | 0.92 (0.50, 1.33) |
|  | United Kingdom | 1 | 1.10 | 0.194 |  |  | -0.18 (-0.50, 0.14) |
|  | Denmark | 1 | 0.33 | 0.739 |  |  | -0.05 (-0.31, 0.22) |
|  | France | 1 | 1.62 | 0.106 |  |  | 0.21 (-0.05, 0.48) |
|  | Japan | 1 | 3.87 | 0.000 |  |  | -0.71(-1.07, -0.35) |
|  | United States | 2 | 0.13 | 0.895 | 0.0 | 0.841 | 0.01 (-0.17, 0.20) |
| Age Group | Children (6–9 years) | 4 | 0.71 | 0.478 | 90.2 | 0.00 | 0.31(-0.54, 1.16) |
|  | Pre-adolescents (10–12 years) | 5 | 0.98 | 0.327 | 94.9 | 0.00 | 0.26(-0.26, 0.79) |
|  | Adolescents (13–18 years) | 1 | 0.24 | 0.194 |  |  | 0.04 (-0.30, 0.38) |
| Population Type | Typically developing | 6 | 0.84 | 0.403 | 72.2 | 0.003 | -0.06 (-0.1.7, 0.06) |
|  | Clinical | 4 | 4.35 | 0.194 | 62.8 | 0.045 | 1.09 (0.88, 1.30) |
| Exercise Modality | Acute Exercise | 5 | 0.38 | 0.703 | 82.7 | 0.000 | -0.07 (-0.40, 0.27) |
|  | Chronic Exercise | 5 | 1.30 | 0.052 | 91.6 | 0.000 | 0.58 (-0.01, 1.16) |
| Session Duration | ≤30min/time | 2 | 0.11 | 0.911 | 0.0 | 0.692 | -0.01 (-0.22, 0.20) |
|  | 31-60min/time | 6 | 1.25 | 0.212 | 94.9 | 0.000 | 0.43 (-0.25, 1.11) |
|  | ＞60min/time | 2 | 0.14 | 0.885 | 71.5 | 0.061 | 0.03 (-0.36, 0.42) |
| Inhibitory Control performance (Reaction Time) | | | | | | | |
| Country | China | 2 | 0.23 | 0.816 | 87.8 | 0.004 | -0.12 (-1.15, 0.91) |
|  | United Kingdom | 1 | 0.51 | 0.608 |  |  | -0.07 (-0.44, 0.31) |
|  | Denmark | 1 | 0.34 | 0.735 |  |  | 0.09 (-0.24, 0.41) |
|  | France | 1 | 3.71 | 0.000 |  |  | 0.50 (0.24, 0.70) |
|  | Japan | 1 | 3.58 | 0.000 |  |  | -0.65 (-1.01, -0.30) |
|  | United States | 2 | 4.13 | 0.000 | 0.0 | 0.523 | -0.39 (-0.58, -0.21) |
| Age Group | Children (6–9 years) | 3 | 0.89 | 0.372 | 85.0 | 0.001 | -0.31(-0.98, 0.37) |
|  | Pre-adolescents (10–12 years) | 4 | 0.20 | 0.841 | 87.3 | 0.000 | 0.04 (-0.37, 0.45) |
|  | Adolescents (13–18 years) | 1 | 2.82 | 0.005 |  |  | -0.49 (-0.82, -0.15) |
| Population Type | Typically developing | 6 | 0.87 | 0.385 | 87.4 | 0.000 | -0.16 (-0.51, 0.20) |
|  | Clinical | 2 | 0.23 | 0.816 | 85.4 | 0.000 | -0.12 (-1.15, 0.91) |
| Exercise Modality | Acute Exercise | 5 | 0.89 | 0.376 | 76.9 | 0.002 | -0.14 (-0.46, 0.17) |
|  | Chronic Exercise | 3 | 0.49 | 0.627 | 92.7 | 0.000 | -0.19 (-0.97, 0.58) |
| Session Duration | ≤30min | 2 | 1.35 | 0.176 | 62.1 | 0.104 | -0.28 (-0.70, 0.13) |
|  | 31-60min | 4 | 1.60 | 0.109 | 77.5 | 0.004 | -0.33 (-0.72, 0.07) |
|  | ＞60min | 2 | 1.46 | 0.143 | 73.6 | 0.052 | 0.30 (-0.10, 0.72) |
| Working Memory performance (Accuracy) | | | | | | | |
| Country | China | 1 | 0.186 | 0.063 |  |  | 0.46 (-0.03, 0.93) |
|  | United Kingdom | 1 | 0.32 | 0.750 |  |  | -0.04 (-0.31, 0.22) |
|  | Japan | 1 | 9.20 | 0.000 |  |  | -3.22 (-3.90, -2.53) |
|  | United States | 2 | 3.43 | 0.001 | 95.8 | 0.000 | -0.64 (-1.01, -0.28) |
| Age Group | Children (6–9 years) | 2 | 0.75 | 0.455 | 98.6 | 0.000 | -1.37 (-4.97, 2.23) |
|  | Pre-adolescents (10–12 years) | 2 | 1.14 | 0.256 | 94.9 | 0.000 | -0.42 (-1.14, 0.30) |
|  | Adolescents (13–18 years) | 1 | 1.58 | 0.114 |  |  | -0.38 (-0.86, 0.09) |
| Population Type | Typically developing | 4 | 2.44 | 0.015 | 96.1 | 0.000 | -1.04 (-1.88, -0.20) |
|  | Clinical | 1 | 1.86 | 0.063 |  |  | 0.46 (-0.03, 0.93) |
| Exercise Modality | Acute Exercise | 4 | 1.78 | 0.075 | 96.8 | 0.000 | -0.85 (-1.78, 0.08) |
|  | Chronic Exercise | 1 | 1.58 | 0.114 |  |  | -0.38 (-0.86, 0.09) |
| Session Duration | ≤30min | 1 | 1.58 | 0.114 |  |  | -0.38 (-0.86, 0.09) |
|  | 31-60min | 3 | 1.51 | 0.132 | 97.3 | 0.000 | -1.15 (-2.65, 0.35) |
|  | ＞60min | 1 | 0.32 | 0.750 |  |  | -0.04 (-0.31, 0.22) |
| Working Memory performance (Reaction Time) | | | | | | | |
| Country | China | 1 | 1.33 | 0.183 |  |  | -0.32 (-0.80, 0.15) |
|  | United Kingdom | 1 | 0.98 | 0.328 |  |  | 0.19 (-0.20, 0.57) |
|  | Japan | 1 | 7.89 | 0.000 |  |  | -2.48 (-3.09, -1.86) |
|  | United States | 2 | 0.93 | 0.355 | 74.8 | 0.047 | -0.23 (-0.73, 0.26) |
| Age Group | Children (6–9 years) | 2 | 1.29 | 0.196 | 96.6 | 0.000 | -1.39 (-3.50, 0.72) |
|  | Pre-adolescents (10–12 years) | 2 | 0.47 | 0.635 | 88.5 | 0.003 | -0.15 (-0.77, 0.47) |
|  | Adolescents (13–18 years) | 1 | 0.29 | 0.768 |  |  | 0.07 (-0.40, 0.54) |
| Population Type | Typically developing | 4 | 1.52 | 0.129 | 94.7 | 0.000 | -0.63 (-1.44, 0.18) |
|  | Clinical | 1 | 1.33 | 0.183 |  |  | -0.32 (-0.80, 0.15) |
| Exercise Modality | Acute Exercise | 4 | 1.81 | 0.071 | 94.3 | 0.000 | -0.72 (-1.51, 0.06) |
|  | Chronic Exercise | 1 | 0.29 | 0.768 |  |  | 0.07 (-0.40, 0.54) |
| Session Duration | ≤30min | 1 | 0.29 | 0.768 |  |  | 0.07 (-0.40, 0.54) |
|  | 31-60min | 3 | 1.94 | 0.052 | 95.0 | 0.000 | -1.05 (-2.10, 0.01) |
|  | ＞60min | 1 | 0.98 | 0.328 |  |  | 0.19 (-0.19, 0.57) |

# **Table S9 Ranking of SUCRA (Surface Under the Cumulative Ranking Curve) values for typically developing children and adolescents.**

| Ranking of SUCRA | 1 | 2 | 3 |
| --- | --- | --- | --- |
| Inhibitory Control performance | | | |
| Accuracy | Badminton (91.91%) | Basketball (75.21%) | Control (42.93%) |
| Reaction Time | Tennis (70.16%) | Basketball (63.03%) | Football (52.94%) |
| Working Memory performance | | | |
| Accuracy | Control (88.63%) | Football (60.28%) | Basketball (50.86%) |
| Reaction Time | Tennis (99.99%) | Basketball (58.43%) | Control (33.89%) |
| Cognitive Flexibility performance | | | |
| Accuracy | Control (86.42%) | Football (63.03%) | Tennis (0.05%) |

# **Table S10** **Egger’s publication test.**

| Detection indicators | *p* Value |
| --- | --- |
| Inhibitory Control performance | |
| Accuracy | *p* = 0.440 |
| Reaction Time | *p* = 0.777 |
| Working Memory performance | |
| Accuracy | *p* = 0.014 |
| Reaction Time | *p* = 0.002 |
| Cognitive Flexibility performance | |
| Accuracy | *p* = 0.290 |
| Reaction Time | *p* = 0.016 |

# **Figure S1** **Risk of bias graph.**

A. The risk of bias summary: review authors’ judgements about each risk of bias item for each included study.

B. The risk of bias graph: review authors’ judgements about each risk of bias item presented as percentages across all included 12 studies.


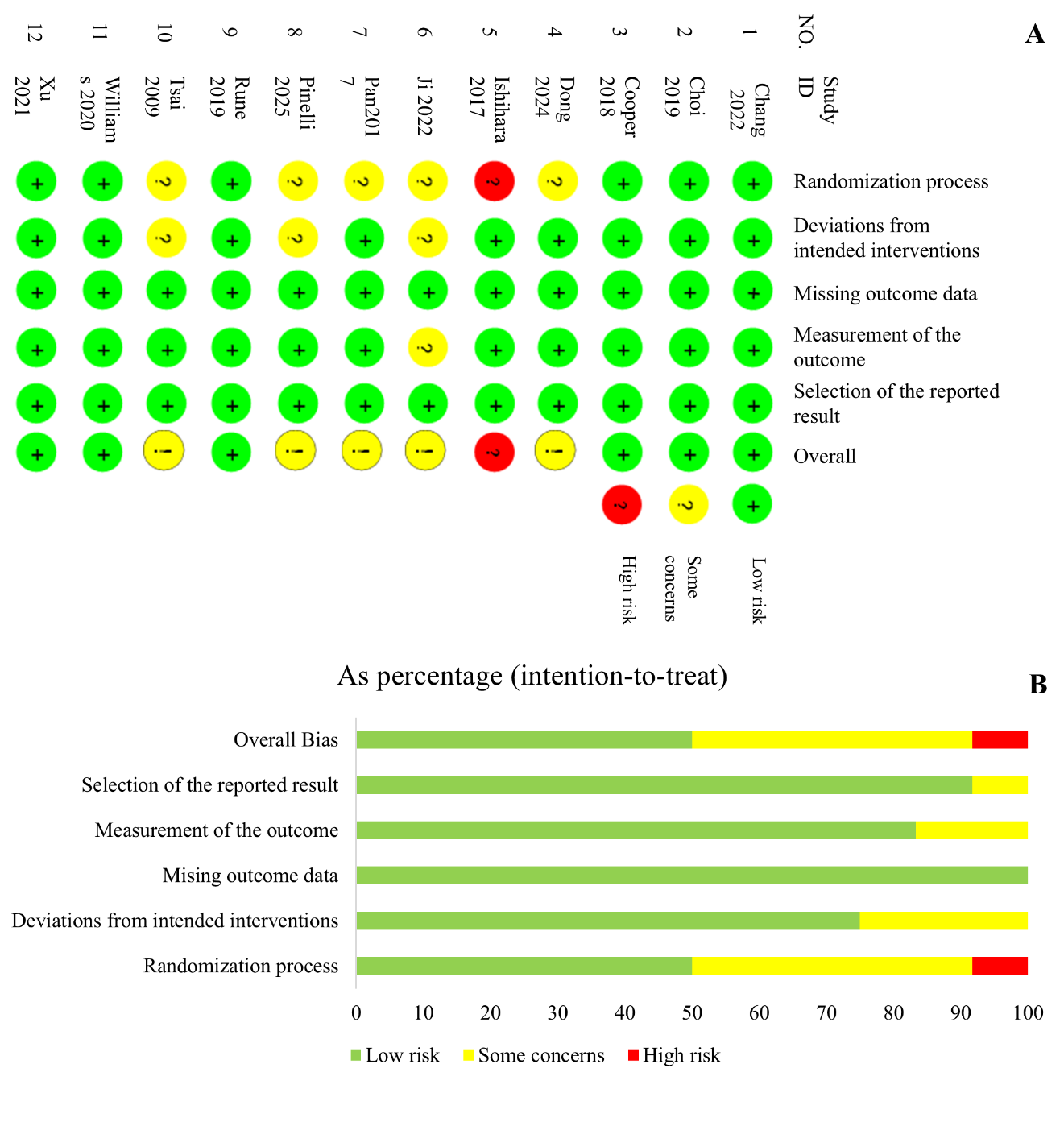


# **Figure S2 Network graph for typically developing children and adolescents.**

Notes: The lines between the dots indicate a direct comparison between the two modes of motion, with thicker lines for more studies and thinner lines for fewer studies.

(A) Accuracy of Inhibitory control, (B) Reaction time of Inhibitory control, (C) Accuracy of working memory, (D) Reaction time of working memory, (E) Accuracy of cognitive flexibility.**
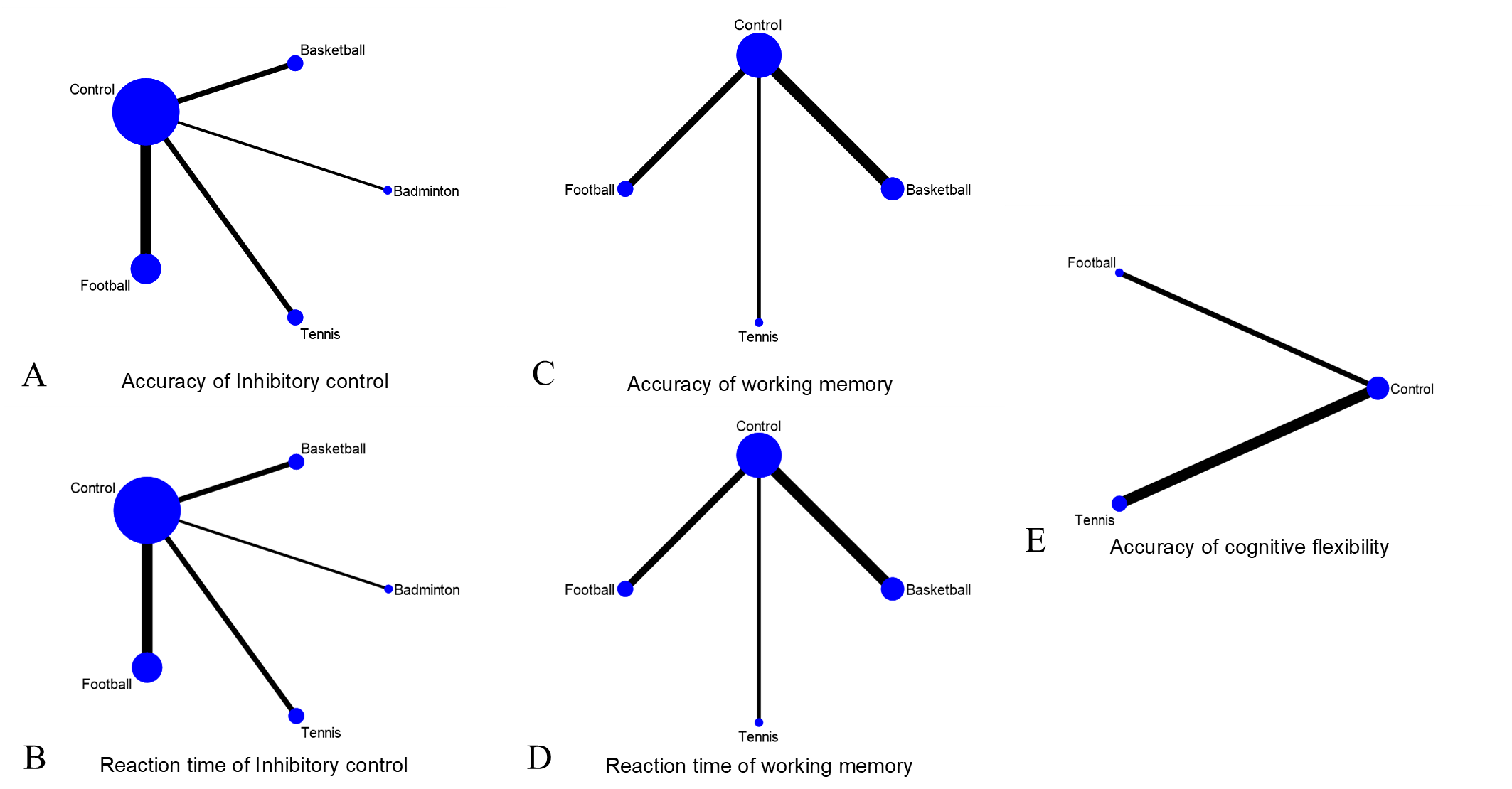
**

# **Figure S3 Leave‑one‑out sensitivity analysis results graph.**

Notes: The horizontal axis represents the effect size. Each dot represents the pooled effect estimate recalculated after omitting the corresponding study, with the whiskers indicating its 95% confidence interval. The three vertical lines represent the overall pooled effect and its confidence interval based on all studies. By comparing the position of each dot relative to the vertical lines, the influence of a single study on the overall results can be assessed. (A) Accuracy of Inhibitory control, (B) Reaction time of Inhibitory control, (C) Accuracy of working memory, (D) Reaction time of working memory, (E) Accuracy of cognitive flexibility.


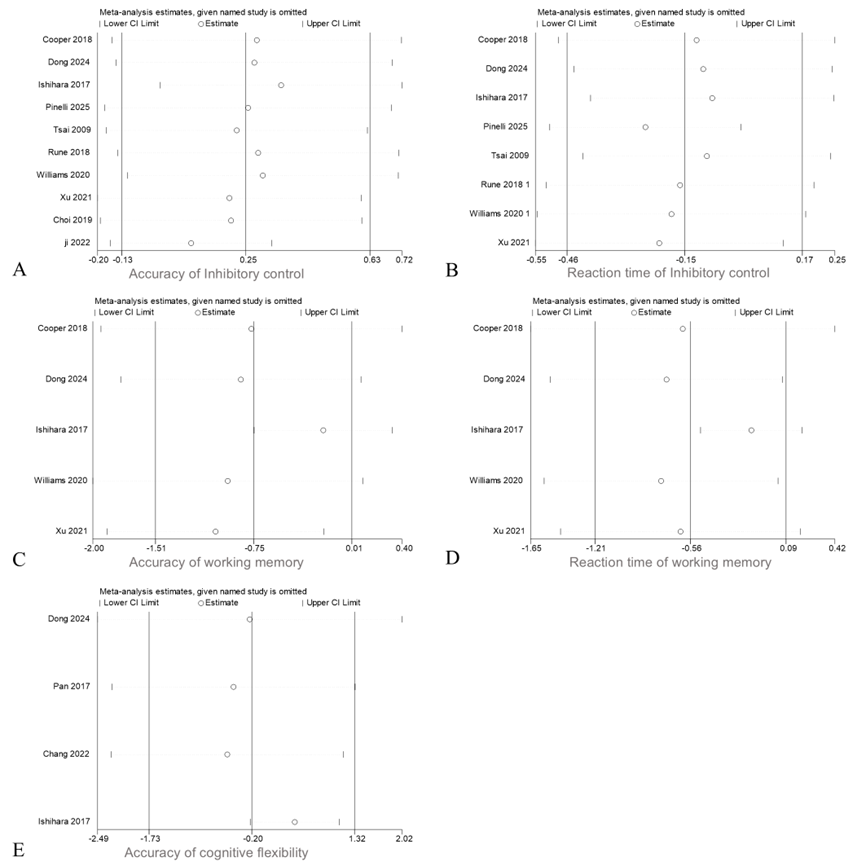

Supplement: Supplementary file 1 [file Table_1.docx]
